# Transcriptome changes in ERGIC3-knockdown hepatocellular carcinoma cells: ERGIC3 is a novel immune function related gene

**DOI:** 10.7717/peerj.13369

**Published:** 2022-05-17

**Authors:** Mengyuan Liu, Qiurong Zhao, Xiang Zheng, Lei Yang, Yanyu Zhao, Xueying Li, Mingsong Wu

**Affiliations:** 1 Zunyi Medical University, Department of Genetics, Guizhou, China; 2 Special Key Laboratory of Oral Disease Research and High Education Institute in Guizhou Province, Guizhou, China

**Keywords:** ERGIC3, Human hepatocellular carcinoma, Cell growth, RNA sequencing, Differentially expressed gene

## Abstract

**Objective:**

The expression of ERGIC3 is increased in a variety of tumors and promotes the growth and metastasis of liver cancer, but the molecular mechanism needs to be further studied.In this study, we aimed to analyze the molecular mechanism of ERGIC3 regulating the proliferation of human hepatocellular carcinoma (HCC) SMMC-7721 cells using transcriptomics.

**Methods:**

ERGIC3 was knocked down in SMMC-7721 cells by RNAi technique, and the expression of ERGIC3 was detected by Q-RT-PCR and Western Blot. RNA sequencing was performed in the Illumina HiSeq platform in the control group and the ERGIC3i group and bioinformatics methods were selected to analyze the data.

**Results:**

The expression of ERGIC3 was reduced to 10% in SMMC-7721 cells by RNAi technique, and 176 genes were up-regulated and 34 genes were down-regulated in ERGIC3i group compared with the control group. Analysis of the pathways and biological processes that enrich the function of differentially expressed genes showed thatthese differentially expressed genes were mainly involved in vesicular transport, growth factors, PI3K-Akt, NOD-like, Jak-STAT, NF-kappa B and other protein kinase-coupled receptors mediated signal transduction pathways, tumor immune response, collagen-integrin receptor-actin axis, and miRNA pathways. More importantly, most of the significantly altered pathways were related to immunity. ERGIC3 may be a key immune-related gene.

**Conclusion:**

Based on the transcriptomic analysis, the mechanism of ERGIC3 promoting the growth of HCC is link with the transport of growth factor receptor, cytokine receptor and collagen. Then it is involved in signal transduction pathways mediated by protein kinase-coupled receptors, PI3K-Akt, NOD-like, Jak-STAT and NF-kappa B. In particular, the mechanism is also involved in the ERGIC3-dependent immune pathways. ERGIC3 is a potential target for prevention and treatment of HCC.

## Introduction

Liver cancer is a common malignant tumor of the digestive tract. The global cancer statistics in 2018 showed that the incidence of liver cancer is the sixth most common malignancy and the mortality is the fourth in the world (Bray et al., 2018). In China, liver cancer is also one of the most deadly cancers (Chen et al., 2016; Chen et al., 2017), posing a serious threat to human health. The occurrence of tumor is the result of the interaction between environmental carcinogenic risk factors and individual genetic factors, and the changes in genetic material play an elementary role in the tumor development. Many genes, such as p53 (Hu et al., 2014), Gal-1 (Leung et al., 2019), Gli2 (Shi et al., 2017), BAG-1 (Gao et al., 2017) and several others, are involved in tumorigenesis of hepatic tumor. Recent studies have found that abnormal high expression of ERGIC3 also promotes the proliferation and migration of HCC cells (Zhang et al., 2013), but the molecular mechanism needs to be further studied. Therefore, this study aimed to investigate the molecular mechanism of ERGIC3 knockdown inhibiting the growth of human hepatocellular carcinoma SMMC-7721 cells based on transcriptomics.

*ERGIC and golgi 3 (ERGIC3)*, also known as *Erv46* and *ERp43*, are located on chromosome 20q11.22, including 14 exons. The encoded protein has a molecular weight of 43.2kDa and is composed of 383 amino acid residues, which is a member of the ERGIC membrane protein family. As a component of COPII vesicle (Otte et al., 2001), it mediates the transport of secreted proteins from rough endoplasmic reticulum to Golgi body, and also contributes to the folding and glycosylation of newly synthesized proteins (Welsh et al., 2006). Inhibition of ERGIC3 expression can affect cell growth and endoplasmic reticulum stress-induced cell death (Wu et al., 2013). In addition, ERGIC3 is a novel tumor-related gene with abnormal expression in a variety of tumors, promoting the growth of HEK-293 cells (Nishikawa et al., 2007), and abnormal expression in non-small cell lung cancer (NSCLC) (Wu et al., 2013; Lin et al., 2015; Hong et al., 2016; Zhao et al., 2020) as well as liver cancer (Zhang et al., 2013) and colorectal tumors (Brim et al., 2014).

Currently, several mechanisms of ERGIC3 in cancer have been reported as follows: (1) Regulating the pathophysiological function of cancer cells through the microRNA pathway.  For example, miR-490-3p modulates cell growth and epithelial to mesenchymal transition of hepatocellular carcinoma cells by targeting ERGIC3 (Zhang et al., 2013). In addition, miR-203a downregulation induces ERGIC3 overexpression in NSCLC cells (Lin et al., 2015). (2) ERGIC3 knockdown suppresses lung cancer through endoplasmic reticulum stress-induced autophagy (Hong et al., 2016). (3) Knocking down ERGIC3 can induce endoplasmic reticulum stress (ERS) in GLC-82 and A549 by up-regulating GRP78, leading to cell cycle arrest (Zhao et al., 2020).

However, the molecular mechanism of ERGIC3 promoting the growth of cancer cells still needs to be further explored. We hypothesized that abnormal expression of ERGIC3 resulted in transport disorder of factors associated with cell proliferation as well as other active biomolecules, which could affect the growth of cancer cells.

Differential gene expression is the molecular basis of tumor heterogeneity, and the study of differentially expressed genes in tumor cells is of great significance for the study of pathophysiological mechanism of tumor genesis and development, while transcriptome sequencing (RNA sequencing, RNA-seq) can reveal the network regulation information of differential gene expression under different physiological and pathological states. Therefore, it is widely used in life science research. The emergence of high-throughput sequencing technology has accelerated the process of understanding the molecular genetics of liver cancer. With the development of a new generation of large-scale sequencing technology, the molecular mechanism of liver cancer will be further explored, so as to more accurately diagnose tumors in the early stage and apply molecular targeted drugs to individualized precise treatment of patients.

## Materials and Methods

### Cell culture

Human hepatocellular carcinoma SMMC-7721 cells, obtained from the Shanghai Cell Bank, Chinese Academy of Sciences, were cultured in complete RPMI 1640 medium (Hyclone, USA) containing 1% penicillin/streptomycin and10% fetal bovine serum (Jiangsu Enmoasai Biotechnology Co., LTD, China) at 5% CO_2_, 37 °C and saturated humidity and subcultured for 2–3 days.

### Experimental grouping and liposome transfection

HCC cells were randomly divided into control group and ERGIC3i group, each group had three biological replicates. One day before transfection, 7.5  × 10^5^ cells per well were seeded in a six-well plate. When the confluence of cells was about 70–80%, Negative Control #1 siRNA and ERGIC3 siRNA (Thermo Fisher Scientific, Waltham, MA, USA) were transfected by Lipofectamine 3000 (Invitrogen, Waltham, MA, USA) into the control group and ERGIC3i group, respectively. The transfection procedures were performed according to the instructions. After 72 h of culture, cell samples were collected for subsequent experiments.

### Total RNA extraction, Q-RT-PCR and library establishment

Total RNA was extracted by TRIzol method, the integrity of total RNA was detected by Gel Doc XR Gel imaging system (Bio-Rad, Hercules, CA, USA), the concentration and purity of total RNA were determined by Nanodrop 2000 ultra-micro spectrophotometer (Thermo Scientific, USA). The cDNA synthesis was performed by according to its specification. Quantitative Real-Time PCR (qRT-PCR) was performed with CFX96 Touch™ fluorescent quantitative PCR instrument (Bio-Rad). The PCR reaction procedure was as follows: 98 °C 30s, 98 °C 5s, 60 °C 5s, 40 cycles. The relative gene expression was calculated by 2^−ΔΔCT^ (Schmittgen & Livak, 2008). The primers sequence were as follows: *GAPDH*, forward: 5′GGCCTCCAAGGAGTAAGACC3′, reverse: 5′AGGGGAGATTCAGTGTGGTG3′; *ERGIC3*, forward: 5′GGAGAGGTACTGAGGACAAATCA3′, reverse: 5′AGCTCATAGAGGACGAAGACTC3′.

Cells in each group were collected, and an appropriate amount of RNAiso Plus lysate (TakaRa Biotechnology, Beijing, China) was added and transported to Shenzhen BGI Co., Ltd on dry ice. Total RNA extraction and quality control, mRNA enrichment, library construction and quality control were completed by Shenzhen BGI Co., Ltd.

### Western blot

The cells were collected, added with RIPA lysis buffer (Solarbio Lifesciences, Beijing, China) and protease inhibitor (BBI, Shanghai, China), and lysed on ice for 10–15 min. The total proteins were obtained by centrifugation, and the protein concentration was determined by BCA method. The proteins were boiled and denaturated, and then separated by SDS-PAGE electrophoresis. The primary antibody (GAPDH (ProteinTech Group, Wuhan, China), 1:10000 dilution, ERGIC3 (Abcam, Cambridge, UK), 1:1000 dilution) was incubated overnight at 4 °C, and the secondary antibody was incubated at 37 °C for 2 h, ECL illuminant was added, and exposure and development were performed.

### Sequencing analysis

Firstly, clean reads were obtained by filtering out low-quality, adaptor-polluted and high content of unknow base (N) reads with SOAPnuke v1.5.2, a filtering software independently developed by BTU. After reads filtering, we mapped those clean reads onto human reference genome GRCh38 using HISAT2 v2.0.4, followed with novel gene prediction. Finally, we identified differentially expressed genes between samples and did clustering analysis and functional annotations.

### Bioinformatics analysis of differentially expressed genes

#### Screening of differentially expressed genes

We detected DEGs with DEseq2 (Fold Change >= 2.00 and Adjusted P value <=0.05) and PossionDis (Fold Change >= 2.00 and FDR <= 0.001) as requested, and the gene expression was calculated using fragments per kilobase per million mapped fragments (FPKM). Using a scatter plot showed the distribution of DEGs, at the same time, using the Draw Venn Diagram (http://bioinformatics.psb.ugent.be/webtools/Venn/) drew the venn diagram. We then performed hierarchical clustering for DEGs using pheatmap, a function of R.

#### GO and KEGG pathway enrichment analysis

With the GO and KEGG annotation result, we classified DEGs according to official classification, and we also performed GO functional enrichment and pathway functional enrichment using phyper in R (FDR <= 0.01) , to determine the main biological functions and pathways of differentially expressed genes.

#### Protein interaction analysis

We mapped the DEGs to the STRING database (http://www.string-db.org) to reveal the protein-protein interaction (PPI) information and visualized the PPI network using Cytoscape 3.8.0. The Analyze Network plug-in was selected to analyze the topology information of PPI network. Subsequently, the MCODE plug-in was conducted to screen modules within PPI network with degree cutoff = 2, node score cutoff = 0.2, k-score = 2 and max.depth = 100. We then analyzed the PPI network to screen the hub genes using the Cytohubba plug-in of Cytoscape, we selected the top 10 DEGs with the highest degrees for further analysis.

#### Survival prognosis analysis of hub genes

The overall survival of 10 key genes were analyzed using the Kaplan-meier (http://kmplot.com/analysis/index.php?p=service&cancer=liver_rnaseq).

### The correlation analysis between ERGIC3 expression and the level of immune cell infiltration

The association between ERGIC3 expression and the abundance of tumor immune infiltrates (B cells, CD8+ T cells, CD4+ T cells, macrophage, neutrophil and myeloid dendritic cells) was analyzed *via* TIMER (http://timer.cistrome.org/) (Li et al., 2020).

### Statistical analysis

The GraphPad Prism 8.3.0 was used for statistical analysis of the qRT-PCR and Western Blot data. The *t*-test was used to compare the mean of two independent samples for intra-group analysis. One-way analysis of variance was used for multi-group comparison. *P* < 0.05 was considered statistically significant.

## Results

### The high expression of ERGIC3 in HCC is associated with survival prognosis

**Figure 1:**
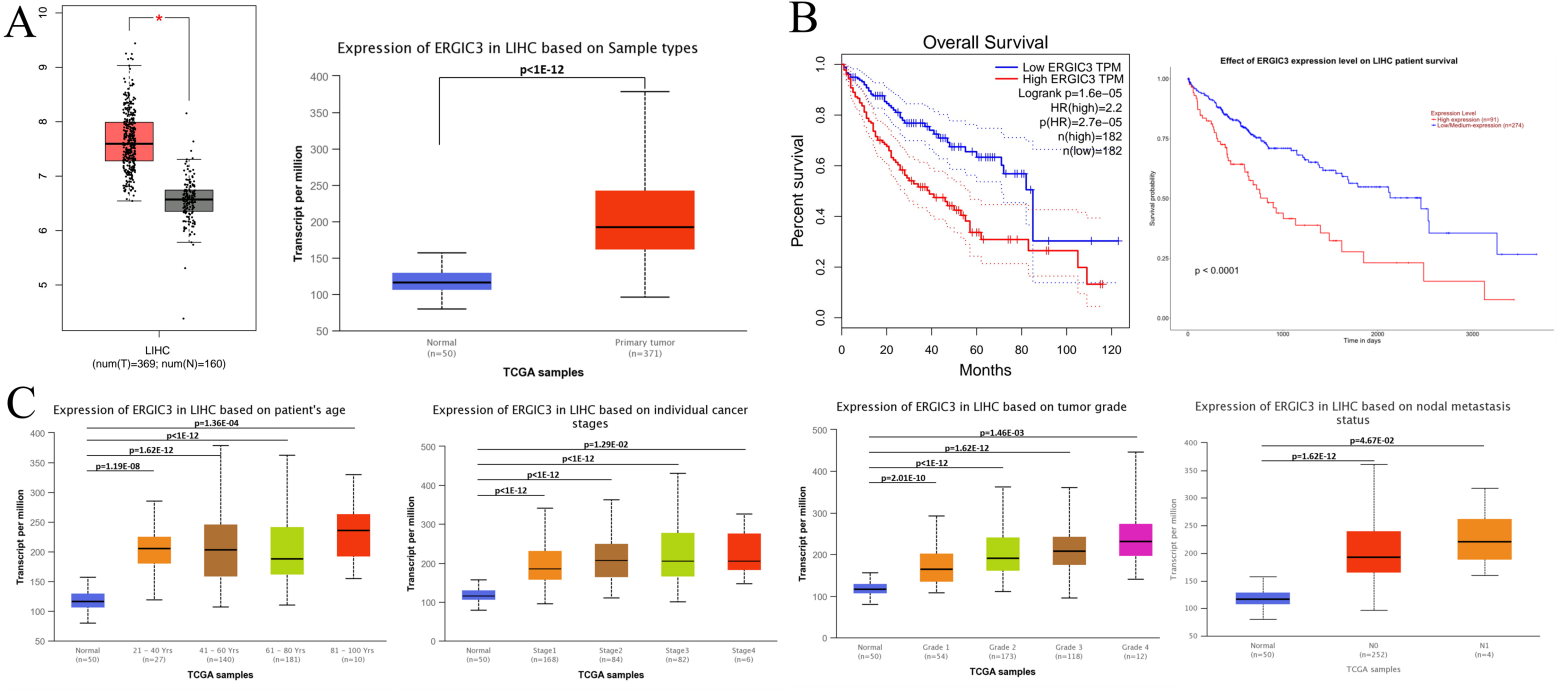
*ERGIC3* levels of liver cancer and its clinical significance. (A) The expression of *ERGIC3* was significantly high in liver hepatoma carcinoma. Tumour tissues represented by red color and normal tissues represented by grey or blue color. (B) Correlation analysis between *ERGIC3* expression and survival of LIHC. (C) The association of ERGIC3 mRNA expression levels with clinical features. Early stage means stage I and stage II; late stage means stage III and stage IV.

Studies have shown that the abnormally high expression of ERGIC3 is associated with the proliferation and growth of HCC cells and epithelial-mesenchymal transition (EMT) (Zhang et al., 2013). Further, GEPIA (http://gepia.cancer-pku.cn/detail.php) database (—log_2_FC ∣cutoff = 1, *p*-value cutoff = 0:01) and UALCAN (http://ualcan.path.uab.edu/analysis.html) online analytical tools were selected to identify the difference of ERGIC3 expression, survival prognosis and its relationship with clinical features between hepatocellular carcinoma patients and normal controls. The results showed that ERGIC3 was highly expressed in HCC (Fig. 1A), and the high expression level of ERGIC3 was associated with poor overall survival rate of HCC patients. The five-year survival rate was about 27.5% (Fig. 1B). In addition, ERGIC3 is more expressed in 61–80 years old, early stage, intermediate T grades, and tumors with No regional lymph node metastasis (Fig. 1C).

### ERGIC3 was successfully knocked down in human hepatoma SMMC-7721 cells

**Figure 2:**
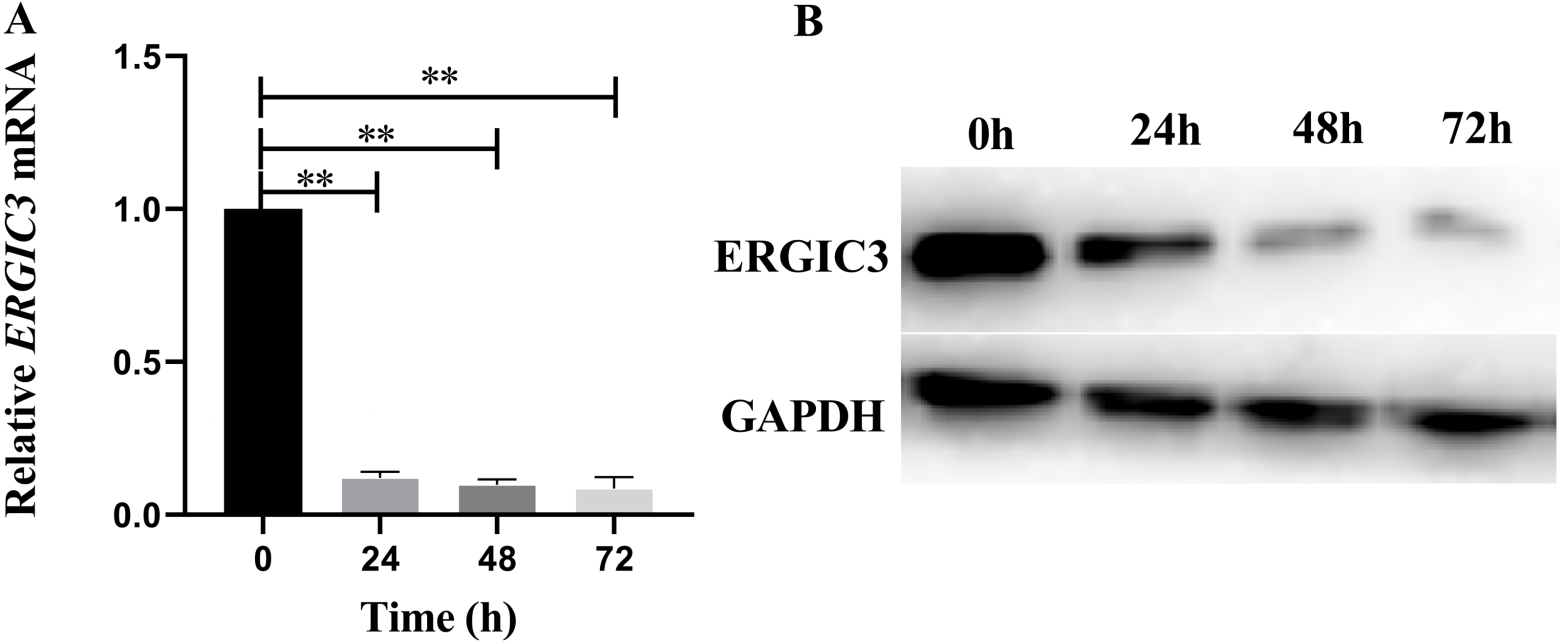
Knockdown of ERGIC3 in SMMC-7721. In SMMC-7721 cells, the expression of ERGIC3 at mRNA (A) and protein (B) levels was significantly reduced after 24 h, 48 h and 72 h treatment with ERGIC3 siRNA. An asterisk (*) indicates significant difference (*P* < 0.05); two asterisks (**) indicate extremely significant difference (*P* < 0.01).

In order to further reveal the molecular mechanism of ERGIC3 promoting the growth of liver cancer cells, ERGIC3 was knocked down in SMMC-7721 cells by RNAi technology. The results of Q-RT-PCR and Western blot analysis demonstrated that ERGIC3 levels was significantly decreased in SMMC-7721 cells after transfection with ERGIC3 siRNA for 24 h, 48 h and 72 h, respectively (Fig. 2). Then transcriptome sequencing technology was used to obtain the differentially expressed genes between the control group and the ERGIC3i group. Finally, the possible molecular mechanisms were analyzed by bioinformatics.

### Sequencing data analysis

RNA sequencing of 6 samples were performed by the Illumina HiSeq platform, and the average output of each sample was 6.90 GB of data. The original transcriptome sequencing data have been submitted to NCBI, the accession number is PRJNA776533. The Q20(%) of clean reads was greater than 98%, and the Q30(%) of clean reads was greater than 95%. Moreover, the average comparison rate of samples for genomes was 85.42% as well as the average comparison rate of the gene set was 71.87%. The above results indicated that it was high sequencing quality (Table 1), and the data could be next step.

**Table 1:** Sequencing data quality inspection and analysis.

Sample	Total raw reads (Mb)	Total clean reads (Mb)	Total clean bases (Gb)	Clean reads Q20 (%)	Clean reads Q30 (%)	Total genome Mapping Ratio (%)	Total gene Mapping Ratio (%)
Acontrol	53.88	46.24	6.94	98.56	95.56	85.19	71.57
AERGIC3i	55.52	47.16	7.07	98.57	95.59	85.85	71.57
Bcontrol	53.80	45.90	6.88	98.69	95.90	85.40	73.23
BERGIC3i	53.75	45.22	6.78	98.62	95.70	85.30	71.48
Ccontrol	53.54	45.04	6.76	98.57	95.59	85.32	71.99
CERGIC3i	55.52	46.43	6.96	98.52	95.46	85.48	71.36

**Figure 3:**
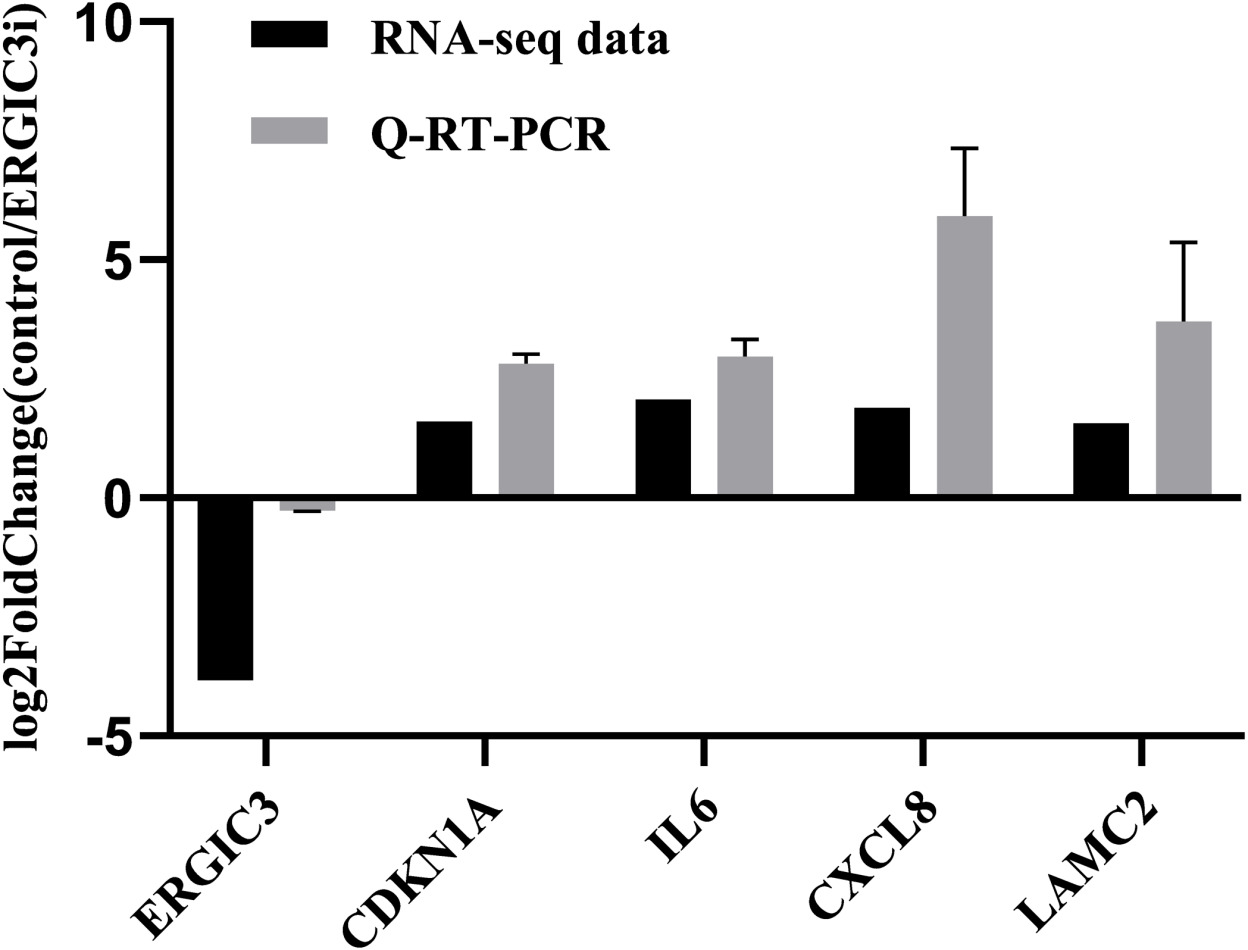
Validation of RNA-Seq data by Q-RT-PCR.

In order to further validate the accuracy of the sequencing results, we randomly selected five genes for Q-RT-PCR analysis. The expression trend of these genes was basically consistent with the transcriptome sequencing results (Fig. 3 and Table 2), which indirectly indicates that the transcriptome sequencing data have a certain credibility.

**Table 2:** Primer sequences used for Q-RT-PCR amplification.

Genes	Sequence (5′–>3′)
ERGIC3	Forward: GGAGAGGTACTGAGGACAAATCA Reverse: AGCTCATAGAGGACGAAGACTC
CDKN1A	Forward: CGATGGAACTTCGACTTTGTCA Reverse:GCACAAGGGTACAAGACAGTG
IL6	Forward: ACTCACCTCTTCAGAACGAATTG Reverse: CCATCTTTGGAAGGTTCAGGTTG
CXCL8	Forward: TCCAAACCTTTCCACCCCAAA Reverse: TTCTCAGCCCTCTTCAAAAACT
LAMC2	Forward: CAAAGGTTCTCTTAGTGCTCGAT Reverse: CACTTGGAGTCTAGCAGTCTCT

### Screening and analysis of differentially expressed genes

**Figure 4:**
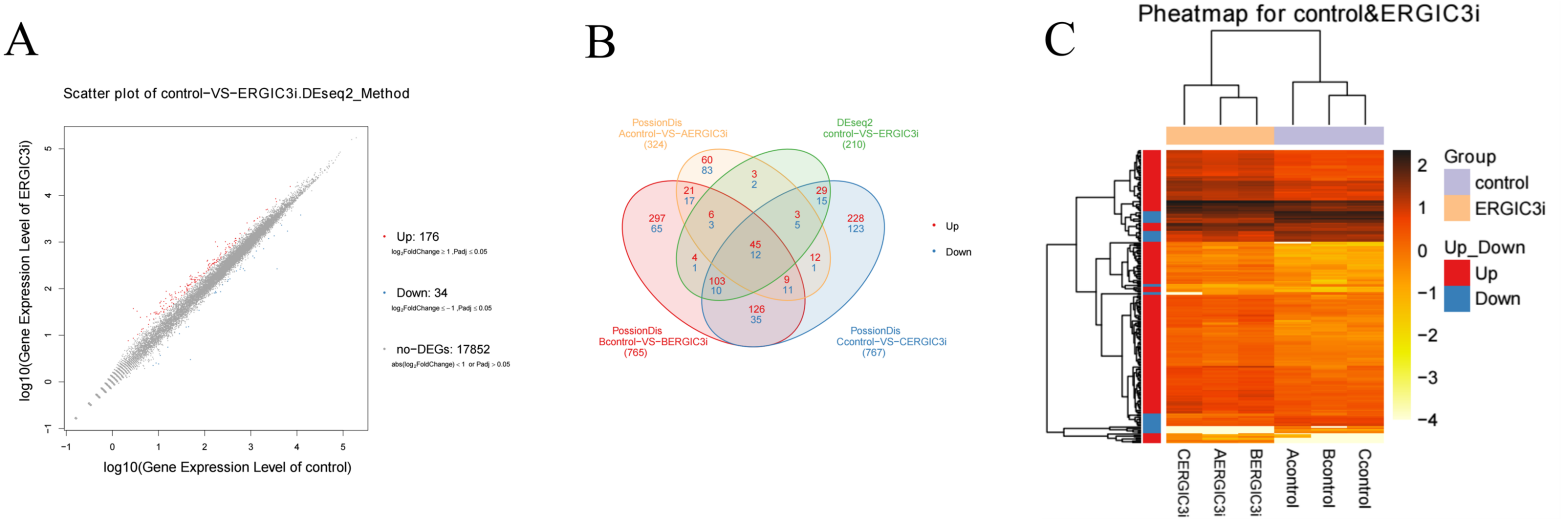
Identification of differentially expressed genes in ERGIC3i group. (A) Distribution of DEGs. The X axis represents the logarithm value of gene expression in the control group, and the Y axis represents the logarithm value of gene expression in the ERGIC3i group. Red represents up-regulated DEGs, blue represents down-regulated DEGs, and gray represents non-DEGs. The criteria for differential gene screening were Fold Change ≥ 2.00 and Adjusted P value ≤ 0.05. (B) Venn diagram of differential results. The number of common and unique DEG between different groups is shown. The red number represents the number of up-regulated genes and the blue number represents the number of down-regulated genes. (C) Pheatmap of DEGs. The X axis represents the samples for cluster analysis, and the Y axis represents the differential genes. The color represents the expression amount converted to log^10^ (the darker the color, the higher the expression amount, the lighter the color, the lower the expression amount).

The differentially expressed genes (DEGs) were defined with FDR<= 0.001 and the difference ratio was more than two fold change. A total of 210 differentially expressed genes were screened, including 192 annotated genes and 18 unknown genes. The expression of each differential gene was plotted on the horizontal and vertical axes according to the values between different groups, namely, as well as the scatter plot of the differential genes was obtained (Fig. 4A). According to the distribution of DEGs, the expression levels of 176 genes were up-regulated and 34 genes were down-regulated. These results indicated that ERGIC3 knockdown resulted in differential expression of many genes in SMMC-7721 cells, suggesting that the promotion of ERGIC3 to HCC cells growth is a complicated process. At the same time, a total of 20 intergroup differential genes were obtained by Venn analysis (Fig. 4B), among which nine genes were up-regulated and 11 genes were down-regulated. In order to more visually display the differentially expressed genes between the control group and the ERGIC3i group, a heatmap was made for each group of DEG (Fig. 4C).

### Go and Pathway enrichment analysis of differentially expressed genes

Gene Ontology (GO) and KEGG database were used to annotate the functions and pathways involved in the differentially expressed genes.

**Figure 5:**
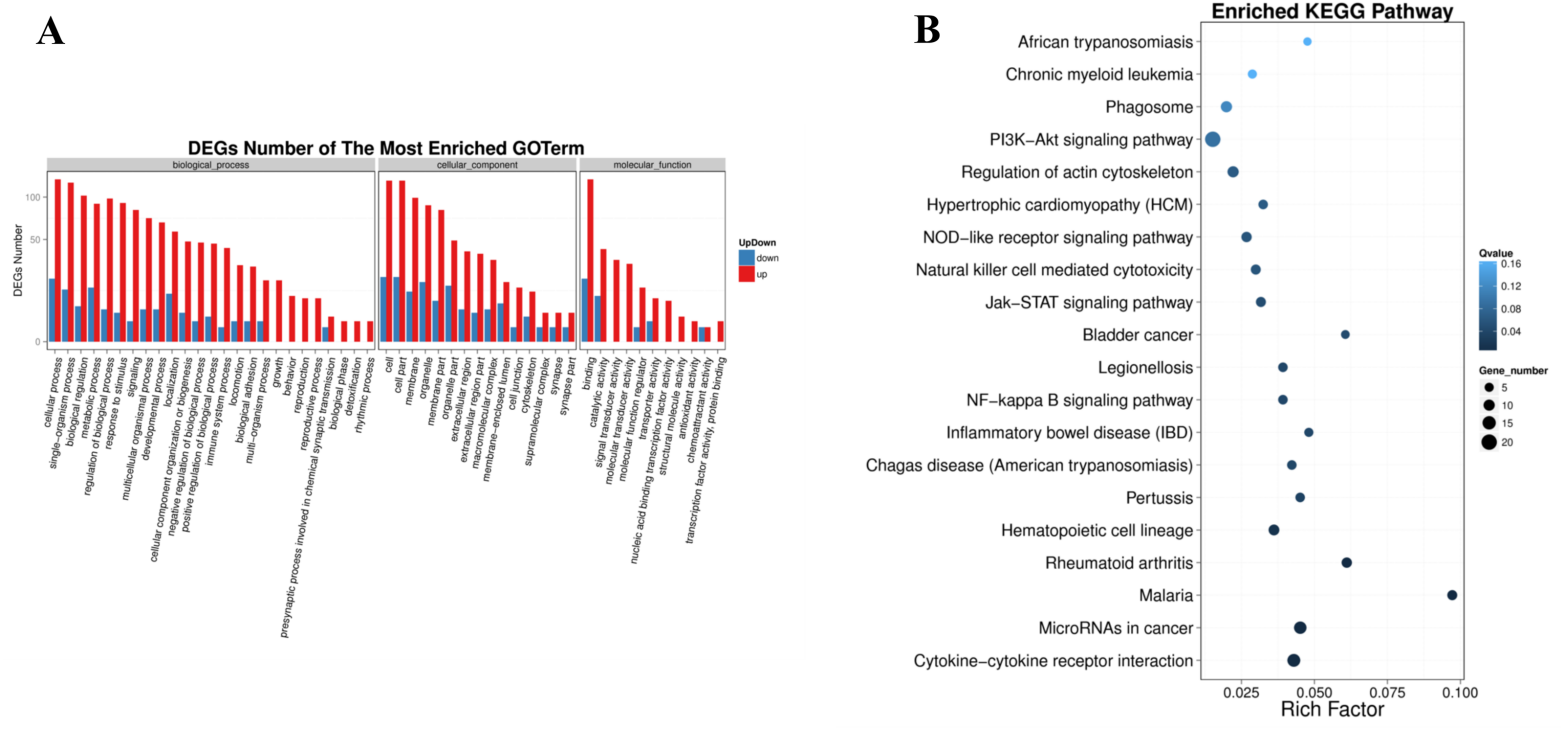
Pathway enrichment of differentially expressed genes. (A) GO classification of up-regulated and down-regulated genes. The X axis represents GO term. The Y axis represents the amount of up/down-regulated genes. (B) Pathway functional enrichment of DEGs. The X axis represents enrichment factor. The Y axis represents pathway name. The color indicates the *q*-value (high: white, low: blue), the lower q-value indicates the more significant enrichment. Point size indicates DEG number (the bigger dots refer to larger amount). Rich Factor refers to the value of enrichment factor, which is the quotient of foreground value (the number of DEGs) and background value (total Gene amount). The larger the value, the more significant enrichment.

It (Fig. 5A) showed that, in the biological process, the most DEGs were involved in biological regulation, metabolic process, signaling, localization, cellular component organization or biogenesis, immune system process, locomotion, biological adhesion, growth. In the cellular component, the DEGs were enriched in macromolecular complex, membrane-enclosed lumen, cell junction, cytoskeleton. Significantly, in the molecular function, the DEGs involved in molecular transducer activity, transporter activity, nucleic acid binding transcription factor activity, transcription factor activity, protein binding and chemoattractant activity.

In the form of directed acyclic graph (DAG), the distribution of enrichment items of different genes at three levels of GO is concentrated, which are biological process (Table S1), cellular component (Table S2) and molecular function (Table S3). The pathway with FDR <=0.01 was considered as significant enrichment. Differentially expressed genes were enriched in the biological process group in regulation of leukocyte migration, positive regulation of leukocyte migration, regulation of signaling, cell surface receptor signaling pathway, positive regulation of leukocyte chemotaxis. The integral component of membrane, extracellular region, extracellular region part, cell periphery, vesicle are significantly enriched in cellular component. Growth factor receptor binding, vascular endothelial growth factor receptor binding, cytokine receptor binding, collagen binding, receptor binding are significant enrichment items in molecular function group.

The significantly enriched pathways were analyzed according to KEGG functional annotation results of differentially expressed genes. The pathway with FDR <= 0.01 was regarded as significant enrichment, and only the top 20 items were shown (Fig. 5B). The enrichment pathway mainly included the PI3K-Akt signaling pathway, focal adhesion, cytokine-cytokine receptor interaction, microRNAs in cancer, phagosome, regulation of actin cytoskeleton, natural killer cell mediated cytotoxicity, chronic myeloid leukemia, NOD-like receptor signaling pathway, Jak-STAT signaling pathway, NF-kappa B signaling pathway. The KEGG functional significance enrichment entries of differentially expressed genes were consistent with the positive regulation of leukocyte migration and chemotaxis, cell surface receptor signaling pathway of biological process components and the growth factor receptor binding, vascular endothelial growth factor receptor binding, cytokine receptor binding, collagen binding of molecular functional components in the GO functional significance enrichment items. It indicated that the related genes regulated by ERGIC3 played an important role in PI3K-Akt, NOD-like, Jak-STAT, NF-kappa B and other protein kinase-coupled receptors mediate signal transduction pathways, tumor nonspecific immune response, collagen-integrin receptor-actin axis, and miRNA pathways. Interestingly, in the top 10 of KEGG pathways of the DEGs, 8 pathways were link to immune system and infectious diseases. The other two were cytokine-cytokine receptor interaction, NF-kappa B signaling pathway, which also played enssential roles in the immune response (Table 3).

### Protein–protein interaction networks of DEGs

**Figure 6:**
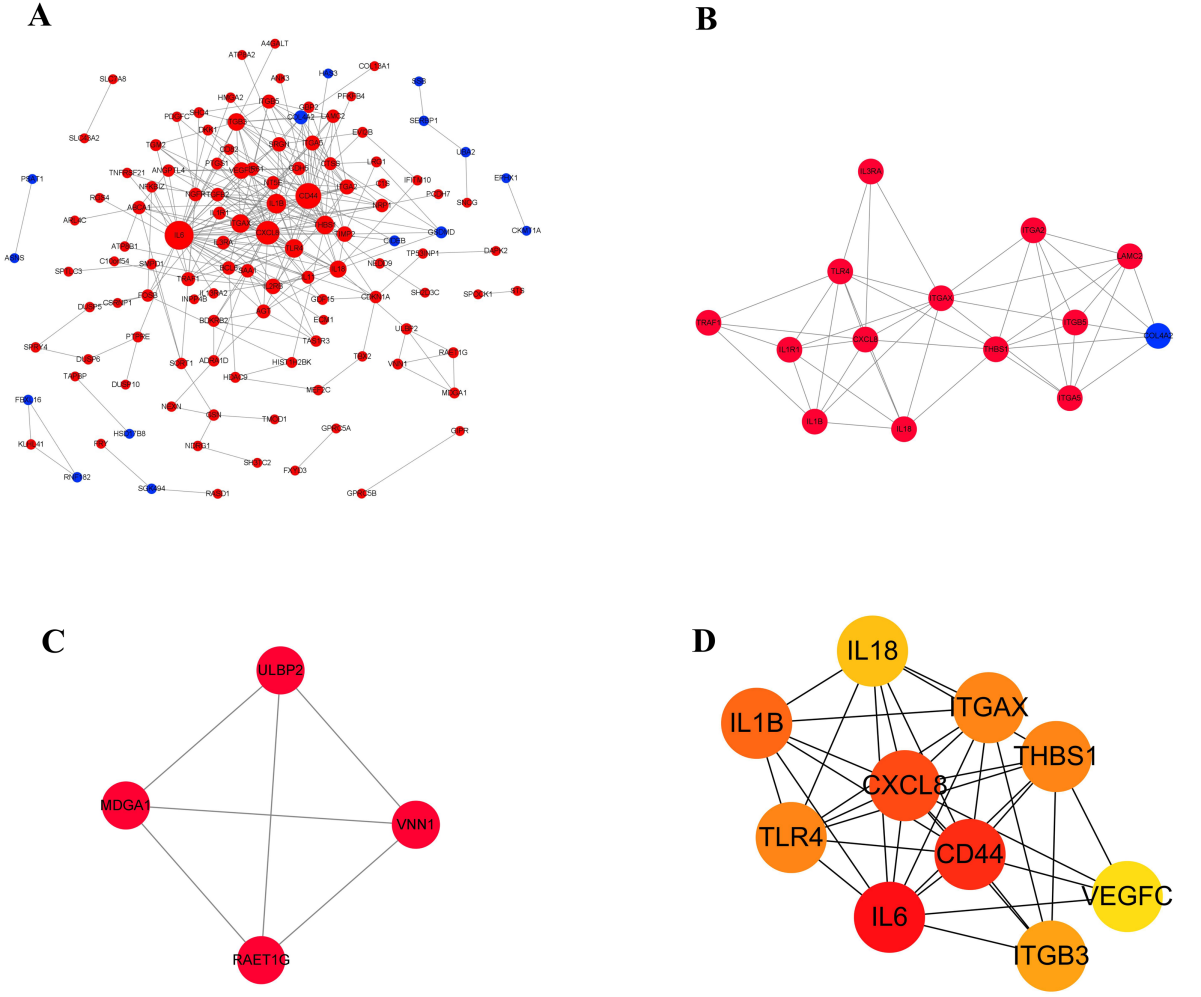
The gene network regulation in the ERGIC3-downregulated cells. (A) Protein-protein interaction network. The nodes of the network are proteins, and the edge represents the interaction between proteins. The red dots refer to up-regulated genes, while the blue dots refer to down-regulated genes. The size of the circle indicates the number of interactions. (B–C) Module analysis. (D) PPI network diagram of hub genes. The color represents the degree value. The redder the color, the greater the degree value.

Proteins usually interact with each other to form complexes that perform specific functions. It is the basis of PPI software and DEGs with interaction usually have similar functions. In order to explore the interaction between 210 differentially expressed genes, a PPI network (Fig. 6A) was constructed using the STRING protein interaction database and Cytoscape software, which revealed 120 nodes and 303 edges in total. Furthermore, MCODE plug-in was used for module analysis, and modules with node number greater than four and MCODE score greater than four were selected to obtain two important modules (Figs. 6B and 6C). It showed that, from the proteins related to modules, the proteins were mainly involved in tumor immune response, integrin gene family and growth factors. Subsequently, in the up-regulated genes, ten hub genes with high connectivity were selected to analysize (Fig. 6D and Table 4). Among these genes, IL6, CD44, and CXCL8/IL8 were the most significant genes with connectivity degrees of 38, 32, 28, respectively. CXCL8 and CD44 were the core in the regulatory network.

**Table 3:** Top10 of KEGG pathways in the DEGs.

Pathway	DEGs genes with pathway annotation (201)	All genes with pathway annotation (24066)	P value	Q value	Pathway ID	Level 1	Level 2
Cytokine-cytokine receptor interaction	14 (6.97%)	326 (1.35%)	6.92E−07	0.000105	ko04060	Environmental Information Processing	Signaling molecules and interaction
MicroRNAs in cancer	13 (6.47%)	288 (1.2%)	1.00E−06	0.000105	ko05206	Human Diseases	Cancers: Overview
Malaria	7 (3.48%)	72 (0.3%)	2.38E−06	0.000166	ko05144	Human Diseases	Infectious diseases: Parasitic
Rheumatoid arthritis	8 (3.98%)	131 (0.54%)	1.48E−05	0.000778	ko05323	Human Diseases	Immune diseases
Hematopoietic cell lineage	9 (4.48%)	249 (1.03%)	0.000259	0.010868	ko04640	Organismal Systems	Immune system
Pertussis	6 (2.99%)	133 (0.55%)	0.000897	0.031396	ko05133	Human Diseases	Infectious diseases: Bacterial
Chagas disease (American trypanosomiasis)	6 (2.99%)	142 (0.59%)	0.001258	0.037752	ko05142	Human Diseases	Infectious diseases: Parasitic
Inflammatory bowel disease (IBD)	5 (2.49%)	104 (0.43%)	0.001822	0.038648	ko05321	Human Diseases	Immune diseases
NF-kappa B signaling pathway	6 (2.99%)	153 (0.64%)	0.00184	0.038648	ko04064	Environmental Information Processing	Signal transduction
Legionellosis	6 (2.99%)	153 (0.64%)	0.00184	0.038648	ko05134	Human Diseases	Infectious diseases: Bacterial

### Survival analysis of hub genes

**Figure 7:**
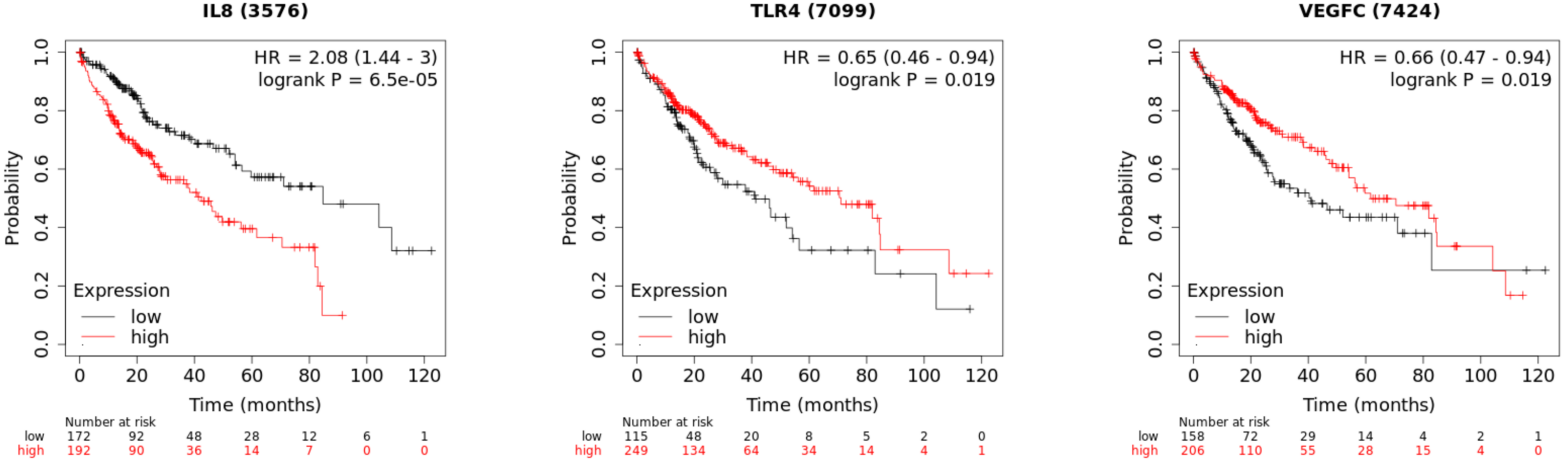
The Kaplan–Meier plots of the core genes.

In order to investigate the prognostic value of the hub genes, Kaplan–Meier was used to analyze the survival data of the 10 hub genes. The results showed that the high level of CXCL8/IL8 was associated with poor overall survival. Patients with high expression of TLR4 and VEGFC had longer overall survival than those with low expression (Fig. 7), while the association of the other seven genes was not significant changes.

### ERGIC3 may be an immune-related key gene

**Figure 8:**
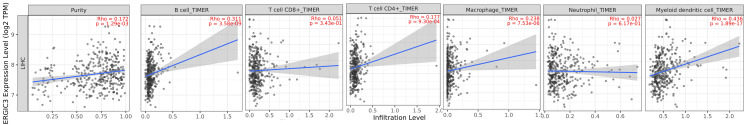
Correlation of ERGIC3 expression with tumor purity and level of immune cell infiltration in HCC. Tumor purity refers to the proportion of tumor cells in a tumor tissues.

Considering that ERGIC3 expression caused significant changes in the interleukin gene family, we speculated that ERGIC3 might be an immune-related key gene. Then we use the TIMER (http://timer.cistrome.org/) algorithm (Li et al., 2020) to evaluate the correlation between ERGIC3 expression and the level of immune cell infiltration. The results demonstrated that the expression of ERGIC3 was positively correlated with the infiltration levels of B cells (*r* = 0.311, *P* < 0.05), macrophage (*r* = 0.238, *P* < 0.05) and myeloid dendritic cells (*r* = 0.436, *P* < 0.05). The expression of ERGIC3 was weakly positively associated with infiltration levels of CD4^+^ T cells (*r* = 0.177, *P* < 0.05), but not CD8^+^ T cells (*r* = 0.051, *P* > 0.05) and neutrophil (*r* = 0.027, *P* > 0.05). Moreover, there was a weak positive correlation between the expression of ERGIC3 and tumor purity (*r* = 0.172, *P* < 0.05) (Fig. 8). These results suggested that ERGIC3 was closely related to immune cell infiltration of HCC.

## Discussion

As a component of vesicle transporter, it is an interesting topic that ERGIC3 indirectly regulates the gene transcription by what it transports. Reports have demonstrated that ERGIC3 is an tumor-related gene and promotes the malignant biological behavior of tumors including hepatocellular carcinoma derived from liver tissue, where metabolism is active and needs a large number of transporter proteins. Disruption of protein transport inevitably leads to dysregulation of transcription factors and indirectly results in changes of gene expression in cells. Thus, transcriptome sequencing and bioinformation was used to investigate the molecular mechanism of ERGIC3 facilitating the growth of hepatoma.

**Table 4:** The top 10 hub genes ranked by degree.

Gene symbol	Gene ID	Gene description	Degree	Regulated	logFC	*P* value
IL6	3569	interleukin 6	38	upregulated	2.0713	3.08E−08
CD44	960	CD44 molecule (Indian blood group)	32	upregulated	1.094103	9.95E−16
CXCL8	3576	*C* − *X* − *C* motif chemokine ligand 8	28	upregulated	1.891171	8.81E−06
IL1B	3553	interleukin 1 beta	20	upregulated	1.124914	0.003173
TLR4	7099	toll like receptor 4	18	upregulated	1.083854	3.86E−05
ITGAX	3687	integrin subunit alpha X	18	upregulated	1.277603	0.003138
THBS1	7057	thrombospondin 1	18	upregulated	1.340374	0.001879
ITGB3	3690	integrin subunit beta 3	16	upregulated	1.993454	1.16E−05
IL18	3606	interleukin 18	15	upregulated	1.092533	1.28E−08
VEGFC	7424	vascular endothelial growth factor C	14	upregulated	1.010739	5.37E−08

Previous reports found that ERGIC3, one of the ERGIC membrane protein family, is mainly involved in the vesicle transport of proteins (Otte et al., 2001; Welsh et al., 2006; Nishikawa et al., 2007; Appenzeller-Herzog & Hauri, 2006). Our sequencing results are also enriched in the integral component of membrane and vesicle. Therefore, it is speculated that the abnormal expression of ERGIC3 may affect cell proliferation and migration by regulating the intracellular transport of proteins. Meanwhile, our results were significantly enriched in the molecular function grouping in growth factor receptor binding, vascular endothelial growth factor receptor binding, cytokine receptor binding, collagen binding. Accordingly, ERGIC3 may regulate the hepatoma growth by the transport of growth factors such as VEGF receptor, cytokine receptor and collagen.

The results were mainly concentrated in the cell surface receptor signaling pathway in the biological process grouping. There are three kinds of cell surface receptor signaling pathways that transmit extracellular mitosis, namely ion channel type, G protein-linked receptor type and enzyme- linked receptor type (Lu et al., 2016). The PI3K-Akt signaling pathway, NOD-like receptor signaling pathway and Jak-STAT signaling pathway as well as NF-kappa B signaling pathway were the most important pathways by KEGG functional annotation analysis. In addition, PI3K-Akt, NOD-like receptor, Jak-STAT, NF-kappa B are all classical signal transduction pathways mediated by protein kinase-coupled receptors. Therefore, we speculate that one of the possible mechanisms of ERGIC3 knockdown inhibiting the growth of HCC is that ERGIC3 down-ragulation reduced the transport of growth factor receptor, VEGF receptor and cytokine receptor, and blocked the signal transduction pathway mediated by protein kinase-coupled receptors such as PI3K-Akt, NOD-like, Jak-STAT, NF-kappa B, as well as slowed down cell cycle.

The occurrence and development of liver cancer is regulated by the immune system, and a lot of important immune molecules, CD44 (Yan et al., 2019; Dhar et al., 2018), IL-6 (Naugler et al., 2007; Wang et al., 2016), CLCX8 (Xiao et al., 2015; Yang et al., 2020). In the GO term, it was also significantly enriched in the positive regulation of leukocyte migration and chemotaxis. Unexpectedly, in the top 10 of KEGG pathways, nine pathways including NF-kappa B were link to immune system and infectious diseases. Moreover, pathway enrichment results were also significantly enriched in the cytokine-cytokine receptor interaction, phagosome, natural killer cells mediated cytotoxicity, chronic myeloid leukemia. In addition, the high expression of ERGIC3 was positively correlated with the infiltration of B cells, macrophage, myeloid dendritic cells, CD4^+^ T and other immune cells, suggesting that ERGIC3 may affect the prognosis of liver cancer patients by raising the abundance of infiltrating immune cells.

Our results reveled that the DEGs were significantly enriched in collagen , focal adhesion and regulation of actin cytoskeleton. In addition, modular analysis of the PPI network using MCODE plug-in found that the protein related to module was mainly involved the integrin family. Integrins are a class of important cell surface receptors that mediate cell-extracellular matrix (ECM) and cell–cell adhesion (Huang, Lin & Chen, 2021; Howden et al., 2021). During cell migration, integrins are involved in the formation of adhesion plaques, and their extracellular domains bind to extracellular matrix such as collagen, fibronectin and laminin, and their intracellular domains connect to cytoskeleton proteins such as *α*-actinin, vinculin and talin, through which they finally connect to actin. Adherent plaques of different shapes and sizes are formed to mediate the forward migration of cells (Howden et al., 2021). Therefore, we hypothesized that ERGIC3 might regulate the proliferation and adhesion of HCC through the collagen-integrin receptor-actin axis.

Our results were also significantly enriched in microRNAs in cancer. Previous studies found that miR-490-3p regulates the growth and EMT of hepatocellular carcinoma cells by targeting ERGIC3 (Zhang et al., 2013). In addition, downregulation of miR-203a induced ERGIC3 overexpression in NSCLC cells (Lin et al., 2015), which was also consistent with previous reports. Therefore, we speculated that ERGIC3 knockdown may inhibit the proliferation and metastasis of human hepatocellular carcinoma SMMC-7721 cells by affecting the miRNA pathway.

Although the possible function of ERGIC3 has been pointed out in our study, and some of the results of previous studies are consistent with our analysis, the reliability of the results requires further experimental verification, and future studies will also verify these predictions at the molecular, cellular and animal levels. In particular, we found that the function of ERGIC3 was link with inflammatory immune response and we will further explore it in the direction.

## Conclusion

ERGIC3, as a member of the ERGIC membrane protein family, activates signal transduction pathways mediated by protein kinase-coupled receptors such as PI3K-Akt, NOD-like, Jak-STAT and NF-kappa B through the transport of growth factor receptor, cytokine receptor and collagen. It affects the abundance of infiltrating immune cells, the collagen-integrin receptor-actin axis and the miRNA pathway to promote the growth of HCC cells.

##  Supplemental Information

10.7717/peerj.13369/supp-1 Supplemental Information 1 Raw data of validation of RNA-Seq data by Q-RT-PCR

10.7717/peerj.13369/supp-2 Supplemental Information 2 Top 10 terms from the biological process Ontology

10.7717/peerj.13369/supp-3 Supplemental Information 3 Top 10 terms from the cellular component Ontology

10.7717/peerj.13369/supp-4 Supplemental Information 4 Top 10 terms from the molecular function Ontology

10.7717/peerj.13369/supp-5 Supplemental Information 5 STR detection report of SMMC-7721 cells The STR test report indicated that the cells we used were SMMC-7721 cells.

10.7717/peerj.13369/supp-6 Supplemental Information 6 The raw data of Q-RT-PCR

10.7717/peerj.13369/supp-7 Supplemental Information 7 The first original GAPDH band taken by Gel Doc XR system during western blotting experiment

10.7717/peerj.13369/supp-8 Supplemental Information 8 The second original GAPDH band taken by Gel Doc XR system during western blotting experiment

10.7717/peerj.13369/supp-9 Supplemental Information 9 The third original GAPDH band taken by Gel Doc XR system during western blotting experiment

10.7717/peerj.13369/supp-10 Supplemental Information 10 The first original ERGIC3 band taken by Gel Doc XR system during western blotting experiment

10.7717/peerj.13369/supp-11 Supplemental Information 11 The second original ERGIC3 band taken by Gel Doc XR system during western blotting experiment

10.7717/peerj.13369/supp-12 Supplemental Information 12 The third original ERGIC3 band taken by Gel Doc XR system during western blotting experiment

## Additional Information and Declarations

### Data Availability

The following information was supplied regarding data availability: The original transcriptome sequencing data is available at NCBI: PRJNA776533. It is also available at FigShare: Wu, Mingsong (2021): the raw data of sequencing data. figshare. Dataset. https://doi.org/10.6084/m9.figshare.16831558.v1.
