# Supplementary material for: Transcriptome changes in ERGIC3-knockdown hepatocellular carcinoma cells: ERGIC3 is a novel immune function related gene
Source: PeerJ. 2022 May 17;10:e13369. doi: 10.7717/peerj.13369 (PMC9121864; doi:10.7717/peerj.13369)
Supplement: Supplemental Information 2 [file peerj-10-13369-s002.docx]

Table S1 Top 10 of terms from the biological process Ontology

| Gene Ontology term | Cluster frequency | Genome frequency of use | Corrected P-value |
| --- | --- | --- | --- |
| [regulation of leukocyte migration](http://amigo.geneontology.org/amigo/term/GO:0002685) | 9 out of 160 genes, 5.6% | 63 out of 16177 genes, 0.4% | 1.36e-05 |
| [positive regulation of leukocyte migration](http://amigo.geneontology.org/amigo/term/GO:0002687) | 8 out of 160 genes, 5.0% | 49 out of 16177 genes, 0.3% | 3.10e-05 |
| [regulation of signaling](http://amigo.geneontology.org/amigo/term/GO:0023051) | 44 out of 160 genes, 27.5% | 1891 out of 16177 genes, 11.7% | 4.31e-05 |
| [cell surface receptor signaling pathway](http://amigo.geneontology.org/amigo/term/GO:0007166) | 44 out of 160 genes, 27.5% | 1921 out of 16177 genes, 11.9% | 6.86e-05 |
| [positive regulation of leukocyte chemotaxis](http://amigo.geneontology.org/amigo/term/GO:0002690) | 7 out of 160 genes, 4.4% | 38 out of 16177 genes, 0.2% | 0.00010 |
| [anatomical structure formation involved in morphogenesis](http://amigo.geneontology.org/amigo/term/GO:0048646) | 22 out of 160 genes, 13.8% | 597 out of 16177 genes, 3.7% | 0.00013 |
| [signaling](http://amigo.geneontology.org/amigo/term/GO:0023052) | 73 out of 160 genes, 45.6% | 4313 out of 16177 genes, 26.7% | 0.00022 |
| [regulation of leukocyte chemotaxis](http://amigo.geneontology.org/amigo/term/GO:0002688) | 7 out of 160 genes, 4.4% | 43 out of 16177 genes, 0.3% | 0.00024 |
| [cell communication](http://amigo.geneontology.org/amigo/term/GO:0007154) | 70 out of 160 genes, 43.8% | 4066 out of 16177 genes, 25.1% | 0.00025 |
| [regulation of cell communication](http://amigo.geneontology.org/amigo/term/GO:0010646) | 42 out of 160 genes, 26.2% | 1876 out of 16177 genes, 11.6% | 0.00028 |
