# Supplementary material for: Transcriptome changes in ERGIC3-knockdown hepatocellular carcinoma cells: ERGIC3 is a novel immune function related gene
Source: PeerJ. 2022 May 17;10:e13369. doi: 10.7717/peerj.13369 (PMC9121864; doi:10.7717/peerj.13369)
Supplement: Supplemental Information 3 [file peerj-10-13369-s003.docx]

Table S2 Top 10 of terms from the cellular component Ontology

| Gene Ontology term | Cluster frequency | Genome frequency of use | Corrected P-value |
| --- | --- | --- | --- |
| [integral component of membrane](http://amigo.geneontology.org/amigo/term/GO:0016021) | 53 out of 176 genes, 30.1% | 2743 out of 17605 genes, 15.6% | 0.00014 |
| [extracellular region](http://amigo.geneontology.org/amigo/term/GO:0005576) | 44 out of 176 genes, 25.0% | 2138 out of 17605 genes, 12.1% | 0.00033 |
| [extracellular region part](http://amigo.geneontology.org/amigo/term/GO:0044421) | 41 out of 176 genes, 23.3% | 2002 out of 17605 genes, 11.4% | 0.00094 |
| [cell periphery](http://amigo.geneontology.org/amigo/term/GO:0071944) | 44 out of 176 genes, 25.0% | 2343 out of 17605 genes, 13.3% | 0.00361 |
| [vesicle](http://amigo.geneontology.org/amigo/term/GO:0031982) | 39 out of 176 genes, 22.2% | 2008 out of 17605 genes, 11.4% | 0.00579 |
| [membrane](http://amigo.geneontology.org/amigo/term/GO:0016020) | 111 out of 176 genes, 63.1% | 8439 out of 17605 genes, 47.9% | 0.00628 |
| [extracellular space](http://amigo.geneontology.org/amigo/term/GO:0005615) | 13 out of 176 genes, 7.4% | 339 out of 17605 genes, 1.9% | 0.00675 |
| [membrane part](http://amigo.geneontology.org/amigo/term/GO:0044425) | 91 out of 176 genes, 51.7% | 6530 out of 17605 genes, 37.1% | 0.00904 |
| [plasma membrane](http://amigo.geneontology.org/amigo/term/GO:0005886) | 42 out of 176 genes, 23.9% | 2280 out of 17605 genes, 13.0% | 0.00929 |
| [secretory granule](http://amigo.geneontology.org/amigo/term/GO:0030141) | 10 out of 176 genes, 5.7% | 216 out of 17605 genes, 1.2% | 0.01159 |
