# Supplementary material for: Transcriptome changes in ERGIC3-knockdown hepatocellular carcinoma cells: ERGIC3 is a novel immune function related gene
Source: PeerJ. 2022 May 17;10:e13369. doi: 10.7717/peerj.13369 (PMC9121864; doi:10.7717/peerj.13369)
Supplement: Supplemental Information 4 [file peerj-10-13369-s004.docx]

Table S3 Top 10 of terms from the molecular function Ontology

| Gene Ontology term | Cluster frequency | Genome frequency of use | Corrected P-value |
| --- | --- | --- | --- |
| [growth factor receptor binding](http://amigo.geneontology.org/amigo/term/GO:0070851) | 8 out of 165 genes, 4.8% | 77 out of 16607 genes, 0.5% | 0.00019 |
| [vascular endothelial growth factor receptor binding](http://amigo.geneontology.org/amigo/term/GO:0005172) | 4 out of 165 genes, 2.4% | 9 out of 16607 genes, 0.1% | 0.00023 |
| [cytokine receptor binding](http://amigo.geneontology.org/amigo/term/GO:0005126) | 11 out of 165 genes, 6.7% | 185 out of 16607 genes, 1.1% | 0.00049 |
| [collagen binding](http://amigo.geneontology.org/amigo/term/GO:0005518) | 4 out of 165 genes, 2.4% | 24 out of 16607 genes, 0.1% | 0.01752 |
| [receptor binding](http://amigo.geneontology.org/amigo/term/GO:0005102) | 26 out of 165 genes, 15.8% | 1168 out of 16607 genes, 7.0% | 0.01768 |
| [protein binding](http://amigo.geneontology.org/amigo/term/GO:0005515) | 92 out of 165 genes, 55.8% | 6894 out of 16607 genes, 41.5% | 0.03004 |
| [collagen receptor activity](http://amigo.geneontology.org/amigo/term/GO:0038064) | 2 out of 165 genes, 1.2% | 3 out of 16607 genes, 0.0% | 0.05995 |
| [TAP2 binding](http://amigo.geneontology.org/amigo/term/GO:0046979) | 2 out of 165 genes, 1.2% | 3 out of 16607 genes, 0.0% | 0.05995 |
| [growth factor binding](http://amigo.geneontology.org/amigo/term/GO:0019838) | 6 out of 165 genes, 3.6% | 99 out of 16607 genes, 0.6% | 0.09415 |
| protein tyrosine/serine/threonine [phosphatase activity](http://amigo.geneontology.org/amigo/term/GO:0008138) | 4 out of 165 genes, 2.4% | 37 out of 16607 genes, 0.2% | 0.09851 |
