# Supplementary material for: Transcriptome changes in ERGIC3-knockdown hepatocellular carcinoma cells: ERGIC3 is a novel immune function related gene
Source: PeerJ. 2022 May 17;10:e13369. doi: 10.7717/peerj.13369 (PMC9121864; doi:10.7717/peerj.13369)
Supplement: Supplemental Information 5 — The STR test report indicated that the cells we used were SMMC-7721 cells. [file peerj-10-13369-s005.pdf]

细胞 STR 检测报告

一、检测样品

样品名称：SMMC-7721

样品编号：BNCC 338089

二、检测项目：STR 基因型检验

三、检测方法：用 Axygen 的基因组抽提试剂盒提取 DNA，采用 21-STR 扩增方案扩增，在 ABI 3730XL 型遗传分析仪上对 STR 位点和性别基因 Amelogenin 进行检测。

四、检验结果：

1，检验基本情况

编号	多等位基因	匹配细胞系	细胞库	EV 值	匹配说明
338089	有	SMMC7721	CRC	1.00	完全匹配

多等位基因指三等位及以上基因现象。

本次检测各细胞分型结果良好。

2，样本描述

BNCC 338089：该株细胞 DNA 分型在细胞系检索中找到基本匹配的细胞系，CRC 数据库显示细胞名为 SMMC7721 细胞号对应 3111C0001CCC000087。本次检测在该细胞系中发现多等位基因。

备注：待测细胞系与收录于 ATCC, DSMZ, JCRB 和 RIKEN 数据库的细胞系 STR 数据进行比对，未收录于以上细胞库的细胞系将无法匹配。

附表：SMMC7721 细胞的 STR 位点和 Amelogenin 位点的基因分型结果

SMMC-7721 细胞			
Marker	Allele 1	Allele 2	Allele 3
D5S818	12	12	
D13S317	13.3	13.3	
D7S820	12	12	
D16S539	9	10	
VWA	16	17	18
TH01	7	7	
AMEL	X	X	
TPOX	12	12	
CSF1PO	10	10	
D12S391	20	25	
FGA	18	21	
D2S1338	17	17	
D21S11	27	28	
D18S51	13.2	16	
D8S1179	12	12	
D3S1358	15	18	

附图：EXPASY 数据库比对结果

STR profile

Markers:	
Amelogenin	X
CSF1PO	10
D2S1338	17
D3S1358	15,18
D5S818	11,12
D6S1043	18
D7S820	12
D8S1179	12
D12S391	20,25,26
D13S317	13.3
D16S539	9,10
D18S51	16
D19S433	13
D21S11	27,28
FGA	18,21
Penta D	8,15
Penta E	7,17
TH01	7
TPOX	12
vWA	16,18

其他说明

分型方案及位点分布

	方案 1	方案 2	方案 3	方案 4
1	D3S1358	D8S1179	D19S433	AMEL
2	VWA	D21S11	TH01	D1S1656
3	D7S820	D16S539	D13S317	D5S818
4	CSF1PO	D2S1338	TPOX	D12S391
5	PENTAE	PENTAD	D18S51	FGA
6			D6S1043	

签发日期：2019.1.30

Applied Biosystems
GeneMapper ID v3.2

0122

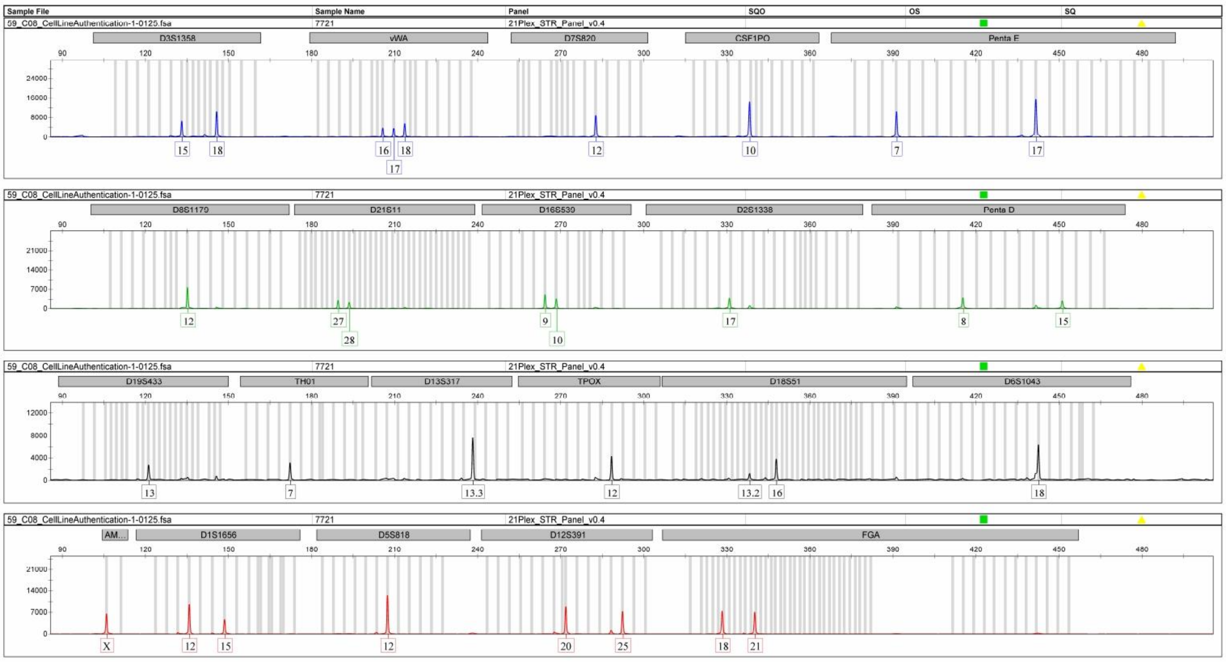

Wed Jan 30,2019 02:11PM, GMT+08:00

Printed by: gmid

关于此细胞株更多信息可在 BNCC 官网查询：www.bncc.org.cn.或发电子邮件至技术指导邮箱咨询 tech@bncc.org.cn。
